# Correction to: Stimulus whitening improves the efficiency of reverse correlation

**DOI:** 10.3758/s13428-022-01981-7

**Published:** 2022-09-29

**Authors:** Alexis Compton, Benjamin W. Roop, Benjamin Parrell, Adam C. Lammert

**Affiliations:** 1 Biomedical Engineering Department, Worcester Polytechnic Institute, 100 Institute Rd, Worcester, MA 01609 USA; 2 Program of Neuroscience, Worcester Polytechnic Institute, Worcester, MA USA; 3 Department of Communication Sciences and Disorders, University of Wisconsin-Madison, Madison, WI USA

**Correction to: Behavior Research Methods**

10.3758/s13428-022-01946-w

The article ‘Stimulus whitening improves the efficiency of reverse correlation,’ written by Compton et al., was originally published electronically on the publisher’s internet portal on August 29, 2022, without open access. With the author(s)’ decision to opt for Open Choice, the copyright of the article changed to ©The authors, 2022, and the article is forthwith distributed under a Creative Commons Attribution ‘cc by.’

