# Supplementary material for: Stimulus whitening improves the efficiency of reverse correlation
Source: Behav Res Methods. 2022 Aug 29;55(6):3120–8. doi: 10.3758/s13428-022-01946-w (PMC10556169; doi:10.3758/s13428-022-01946-w)

Supplement to "Stimulus Whitening Improves the Efficiency of Reverse Correlation"

Alexis Compton[1], Benjamin W. Roop[2], Benjamin Parrell[3], Adam C. Lammert[1]

[1] Biomedical Engineering Department, Worcester Polytechnic Institute

[2] Program of Neuroscience, Worcester Polytechnic Institute

[3] Department of Communication Sciences and Disorders, University of Wisconsin-Madison

Whereas the present paper discusses the effects of whitening the *rows* of the stimulus matrix X, it is also possible to whiten the *columns* of X. Eq 2 includes the term $(X^T X)^{-1}$, which compensates for covariance among the *columns* of X, and which would have a similar effect to pre-whitening the columns of X. It is common to simplify Eq 2 into Eq 3 by eliminating this term in accordance with the assumption that X is already sufficiently whitened with respect to its columns. Depending on the application context – for instance when *n* is small – it might be ill-advised to use the simplified Eq 3, because correlations in the columns of X may lower the accuracy of the resulting estimate.

With regard to the efficiency of reverse correlation, it is important to note that the effects of whitening the rows of X are not redundant with, and largely unrelated to, those obtained by whitening its columns. In particular, the observed benefit of whitening the rows of X would not be eliminated by whitening the columns of X, or using Eq 2, because whitening the columns does not necessarily whiten the rows, and because it is whitening of the rows that is critical for improving estimation accuracy, as the mathematical justification provided in the main paper explains.

To demonstrate the relative effects of using the full Eq 2 versus implementing the proposed whitening method, we repeated the experiments described in the main paper using Eq 2 instead of Eq 3. These experiments were intended (a) to provide a direct comparison of the estimation quality stemming from reverse correlation with whitened stimuli vs. reverse correlation using Eq 2, as opposed to the simplified Eq 3, and (b) to assess the extent to which our stimulus whitening procedure may be redundant with the benefits provided by using Eq 2.

Fig A reproduces the results shown in Fig 2 from the main paper (at p=900; using Eq 3), but with results using Eq 2 superimposed, and with the relevant comparisons (i.e., the effect of using Eq 2; the effect of stimulus whitening) annotated. Results show, first, that stimulus whitening outperforms column whitening (i.e., using Eq 2) in all cases and, second, that the effect of stimulus whitening is not redundant with the effect of column whitening (i.e., the benefit of stimulus whitening is over and above that provided using Eq 2).

Figure A

Estimation quality (mean and 95% CI) for both random, unwhitened stimuli (dashed lines) and whitened stimuli (solid lines) as a function of number of stimuli presented (n). Estimation quality results are shown for reverse correlation using Eq 3 (i.e., $\hat{\beta} = \frac{1}{p}X^T y$*; gray lines), which is typical in reverse correlation, and using Eq 2 (i.e.,* $\hat{\beta} = (X^T X)^{-1} X y$*; blue lines). The effect of stimulus whitening, and of using Eq 2, are indicated with brackets on the right-hand side of the plot.*

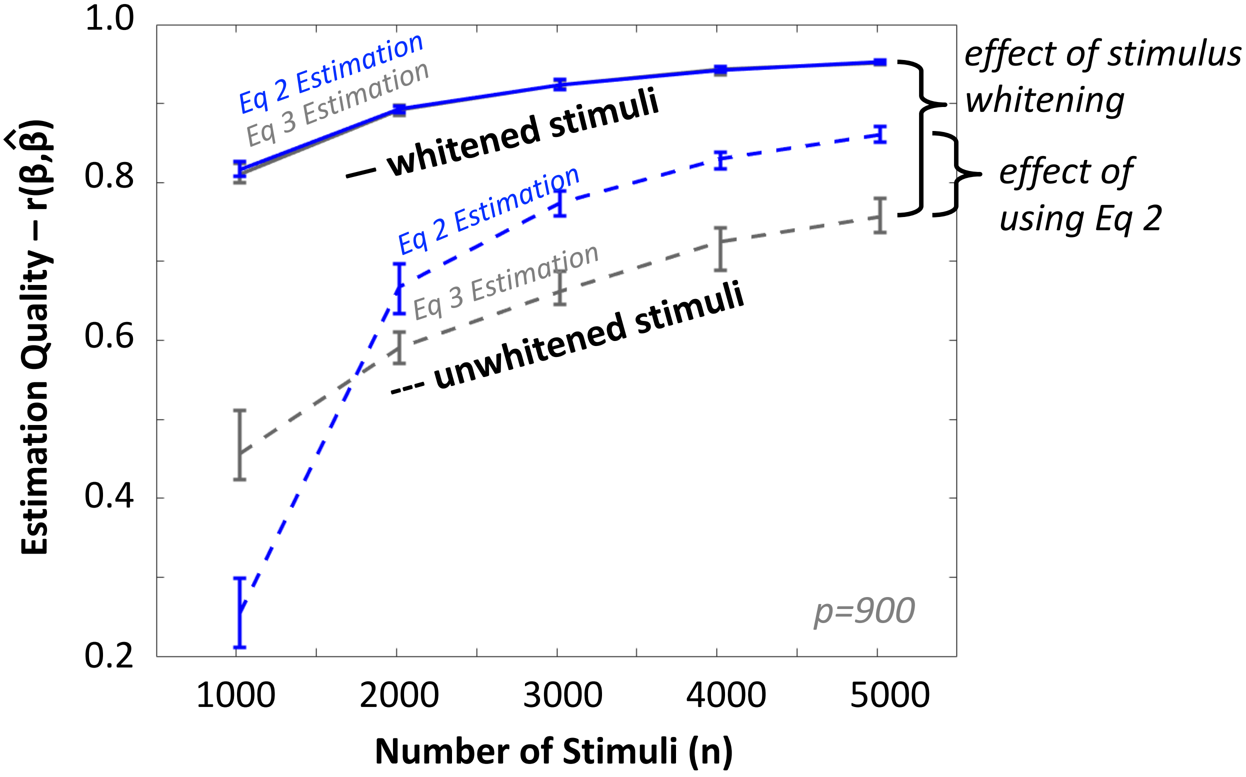

Supplement: Supplementary file 1 — (PDF 407 kb) [file 13428_2022_1946_MOESM1_ESM.pdf]
